# Erratum: Bibliometrics effects of a new paper level classification

**DOI:** 10.3389/frma.2025.1603155

**Published:** 2025-04-10

**Authors:** Frontiers Production Office

**Affiliations:** Frontiers Media SA, Lausanne, Switzerland

**Keywords:** scientometrics, classification systems, Scopus, ASJC, item-by-item classification

Due to a production error, there was a mistake in Figures 1, 2, 4–8 as published. The current figure order is incorrect and has led to a mismatch between the figures and the captions.

The corrected figures appear below.

**Figure 1:**
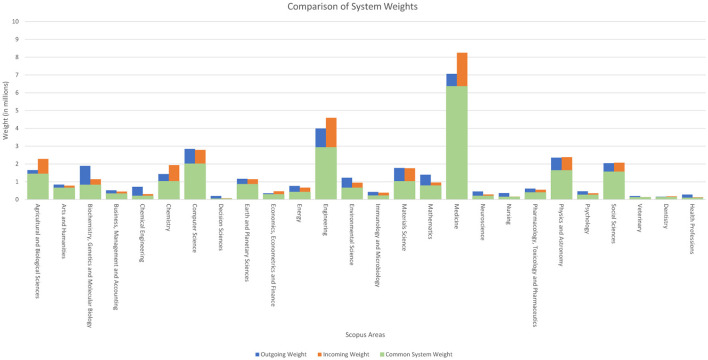
Comparison between the total weights of each Scopus area according to the system used.

**Figure 2:**
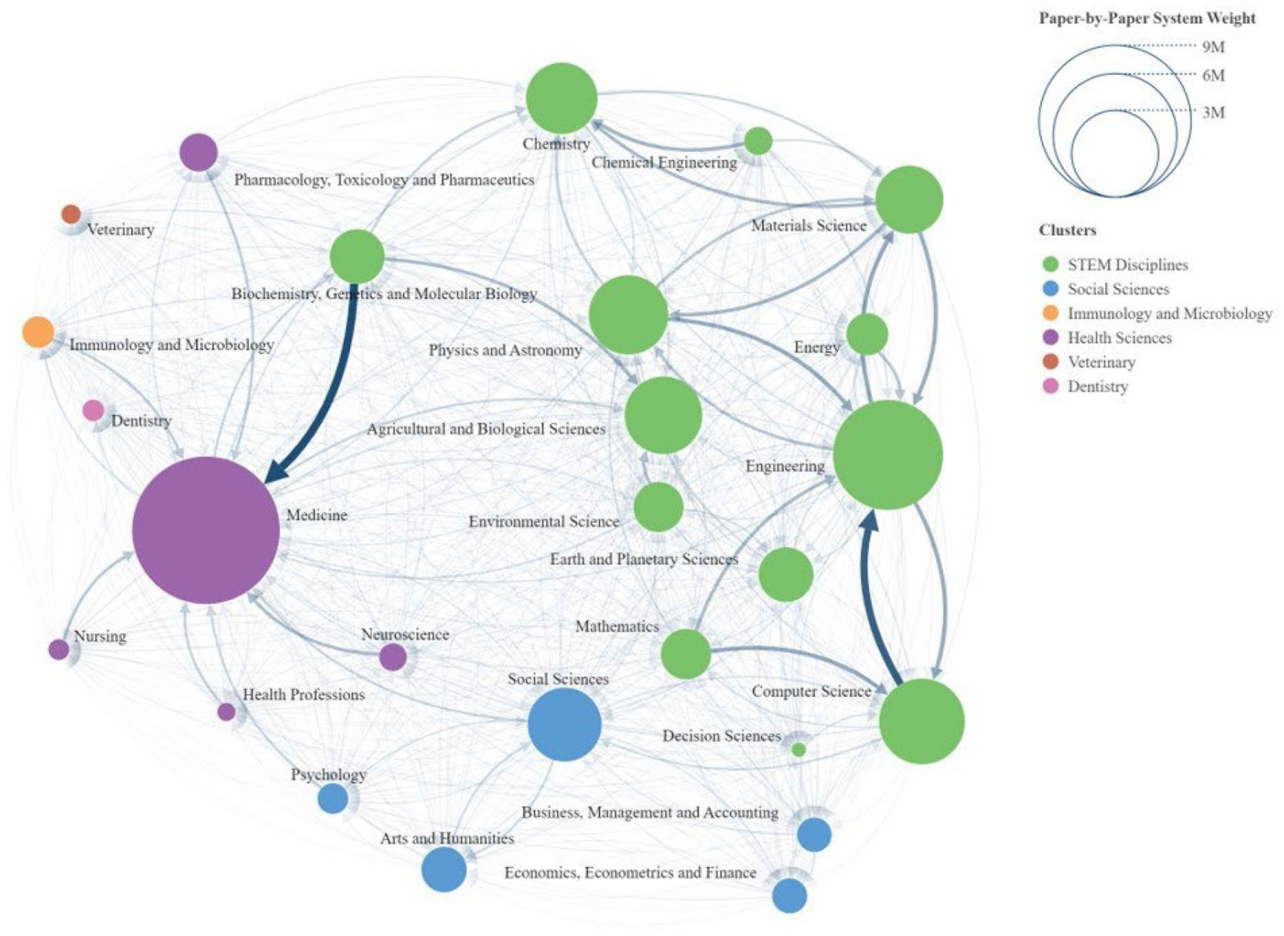
Scopus areas network diagram.

**Figure 4:**
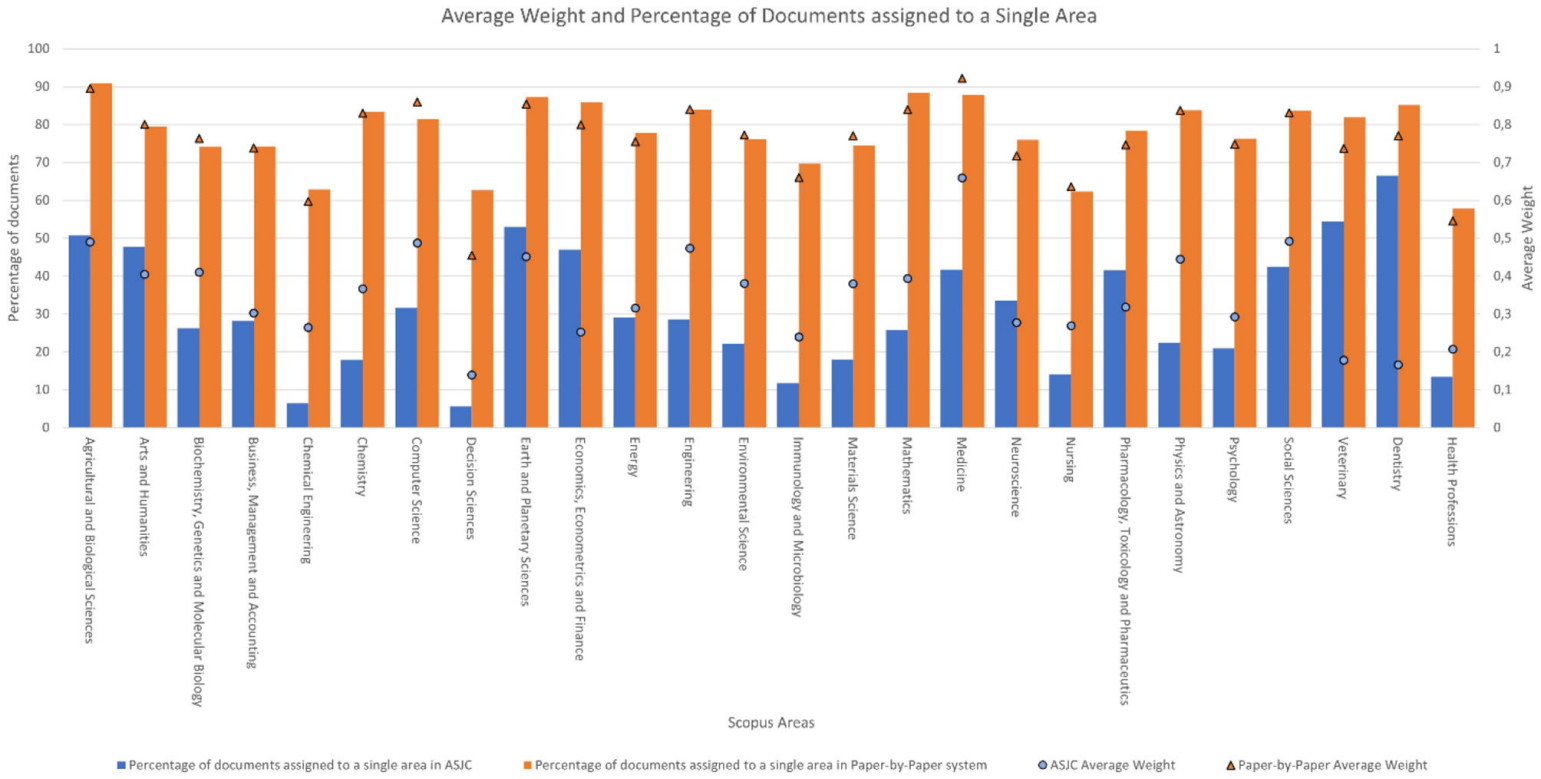
Average weight and percentage of documents assigned to a single area according to the system used.

**Figure 5:**
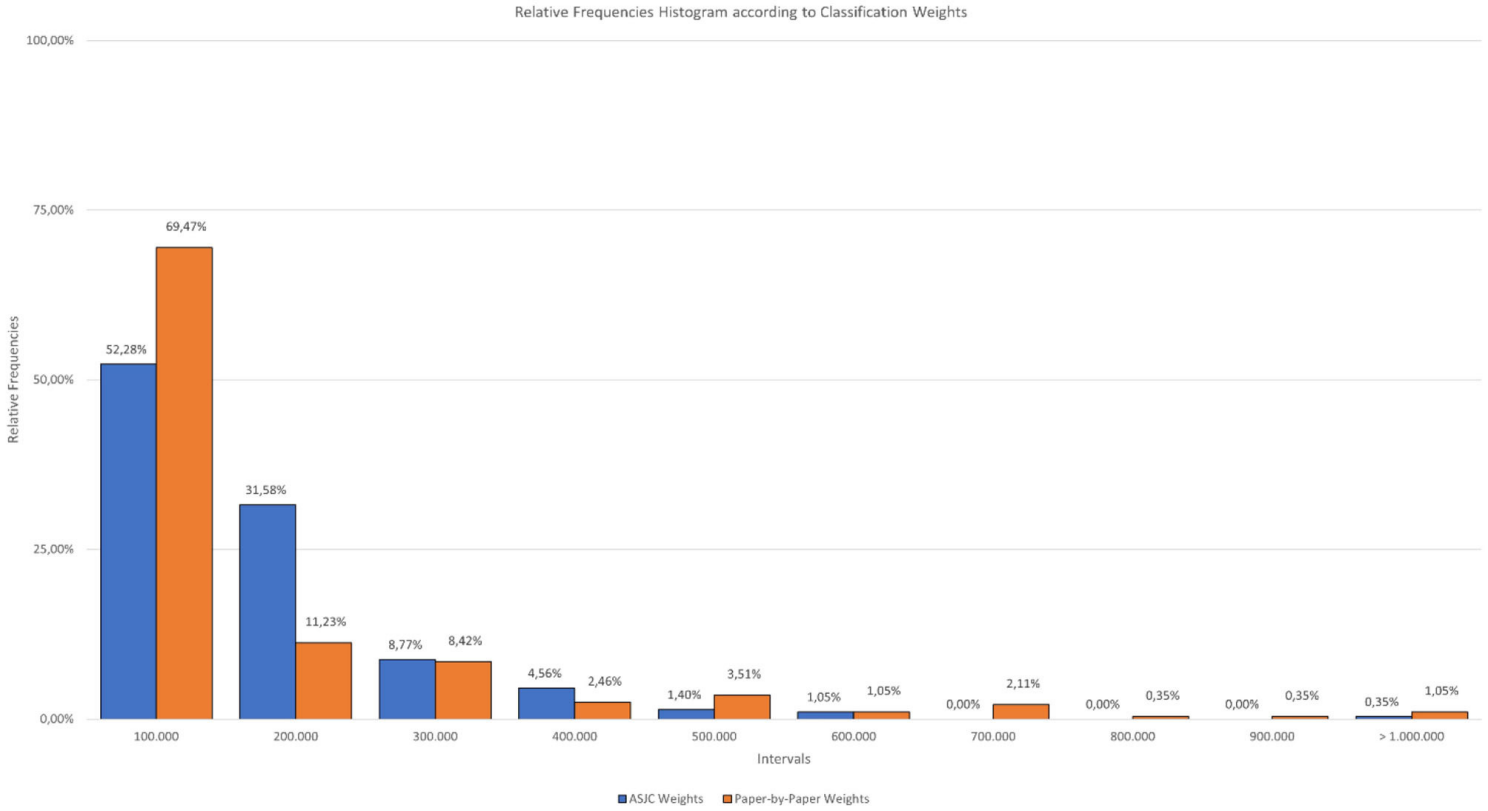
Relative frequencies histogram according to classification weights.

**Figure 6:**
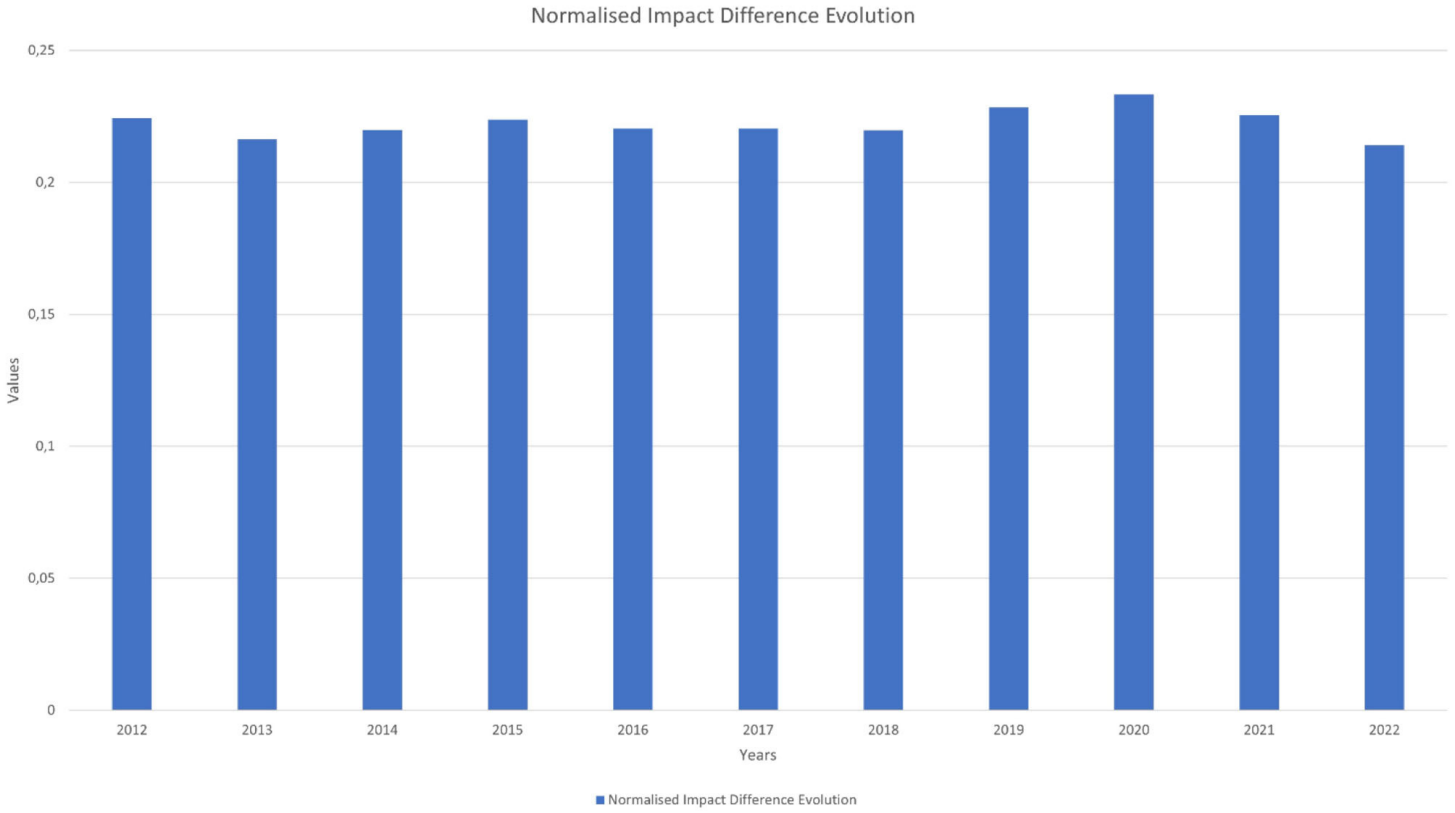
Normalised impact difference evolution.

**Figure 7:**
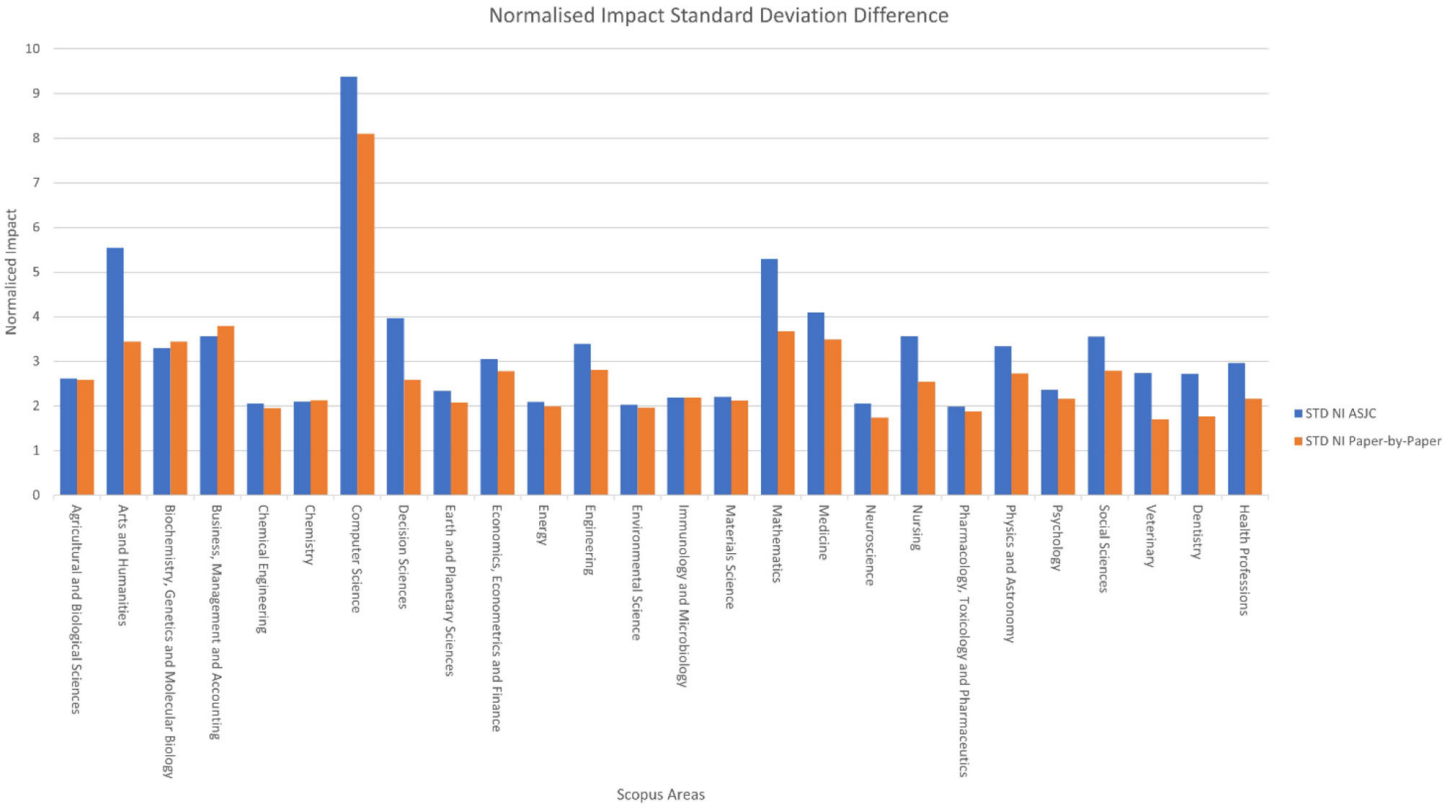
Normalised impact standard deviation difference by area.

**Figure 8:**
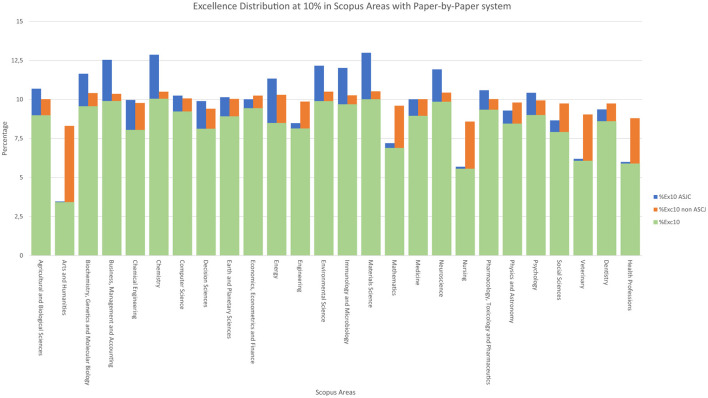
Excellence distribution at 10% in Scopus areas with paper-by-paper system.

The publisher apologizes for this mistake. The original article has been updated.

